# The Effects of Premedication With Three Different Doses of Intravenous Dexmedetomidine on Spinal Anesthesia: A Randomized Comparative Study

**DOI:** 10.7759/cureus.52459

**Published:** 2024-01-17

**Authors:** Chandraleela Sundararajan, Ganesh Singaravelu, Karthikeyan Selvaraj, Sathyasuba Meenakshisundaram, Raghuraman M Sethuraman, Amarnath Moni

**Affiliations:** 1 Anesthesiology, Sri Venkateshwaraa Medical College Hospital, Puducherry, IND; 2 Otorhinolaryngology, Sri Lakshmi Narayana Institute of Medical Sciences, Puducherry, IND; 3 General Surgery, Sree Balaji Medical College & Hospital, Bharath Institute of Higher Education and Research, Chennai, IND; 4 Anesthesiology, Sree Balaji Medical College & Hospital, Bharath Institute of Higher Education and Research, Chennai, IND; 5 Anesthesiology, Chettinad Medical College Hospital and Research Institute, Chennai, IND

**Keywords:** motor block, duration of analgesia, subarachnoid block, intravenous sedation, dexmedetomidine

## Abstract

Background: Intravenous dexmedetomidine is one of the commonly preferred techniques for sedation during any regional procedure. However, only a very few studies compared the impact of different bolus doses during spinal anesthesia, and none for our geographical area.

Materials and methods: A total of 60 patients were allocated into either of the three groups (group I, II, III) to receive intravenous dexmedetomidine 0.5, 0.75, and 1 mcg/kg, respectively. The primary outcome was to compare the duration of sensory and motor blockade and the secondary outcomes were the level of sedation, hemodynamic stability, duration of analgesia, and the side effects, if any.

Results: Two-dermatome regression time and the duration of motor block were significantly higher in groups II and III when compared to group I. However, the difference in duration of analgesia, Ramsay sedation scores, bradycardia, and hypotension was statistically insignificant between the groups.

Conclusion: Intravenous dexmedetomidine in doses of 0.75 and 1 mcg/kg significantly prolongs the two-dermatome regression time and duration of the motor block when compared to 0.5 mcg/kg dose. Hence, it is better to titrate the dose between 0.75 and 1 mcg/kg, as the administration of bolus intravenous Dex in doses ranging between 0.75 and 1 mcg/kg appears to provide adequate intraoperative block characteristics while maintaining hemodynamic stability without any significant respiratory depression or other adverse effects.

## Introduction

The subarachnoid block is the commonly preferred anesthetic technique for infra-umbilical and lower-limb surgeries. However, some patients do not accept it because they do not want to be awake during the surgical procedure due to various factors, including environmental [1], thus mandating the administration of sedative and amnesic drugs.

Several clinical studies, including one meta-analysis, have reported the effects of intravenous dexmedetomidine (Dex) on subarachnoid block [2-13]. While some of the studies analyzed the effects of intravenous Dex by administering it first as a bolus followed by infusion [2-7], other studies analyzed it as either a bolus only [8-10] or as a continuous infusion only [12]. Besides, one study compared the bolus dose versus the infusion of Dex to evaluate the effects on the subarachnoid block [13].

Very few studies compared the impact of different bolus doses of intravenous Dex in this context [8-10]. The optimal dosing is yet to be defined. More incidences of bradycardia and excessive sedation are warranted with a higher dose approaching 1 mcg/kg. Though a review article by Upadhyay [14] concluded that intravenous Dex provides adequate intraoperative sedation, increases patient comfort, improves spinal block quality, and prolongs postoperative analgesia, it instilled in us the quest for optimal dosing for the same. To the best of our knowledge, our study is the first to compare the three different doses (0.5, 0.75, and 1 mcg/kg) of Dex as a bolus intravenous administration before subarachnoid block to find the optimum dose. The primary outcome of the study was to compare the duration of sensory and motor blockade between the three groups. The secondary outcomes were the level of sedation, hemodynamic stability, duration of analgesia, and the side effects, if any.

## Materials and methods

This study was approved by the Institutional Review Board (IRB) and all patients gave written consent to participate in the study. This prospective, randomized, double-blind study was conducted at a tertiary care teaching hospital. The patients scheduled for infra-umbilical and lower limb surgeries under spinal anesthesia, fulfilling the inclusion criteria were chosen to be part of the study.

All patients with the American Society of Anesthesiologists (ASA) physical status grade I or II with ages between 18 and 60 years of either sex were included in the study.

The exclusion criteria were as follows: (1) known allergy to Dex or local anesthetics; (2) patients on sedative medications, opioids, antidepressants, β-blockers, angiotensin-converting enzyme inhibitors (ACEI), angiotensin receptor blockers (ARB), and clonidine; (3) patients with a resting heart rate < 60 bpm, systolic pressure < 100 mmHg, and a BMI < 18.5 or > 30 kg/m2; (4) patients with a history of alcohol or drug abuse; (5) patients with severe chronic obstructive pulmonary disease; (6) any contraindications to spinal anesthesia; and (7) patients with renal and hepatic insufficiency.

Sixty eligible patients were allocated into one of the three groups (each group comprising 20 patients) using a computer-generated randomization code. Group I, group II, and group III patients received intravenous Dex 0.5, 0.75, and 1 mcg/kg, respectively, over 15 minutes just before administration of the subarachnoid block.

After noting the baseline heart rate (HR), systolic blood pressure (SBP), diastolic blood pressure (DBP), oxygen saturation (SpO2), respiratory rate (RR), and Ramsay saturation score [15], all patients received 500 ml of Ringer lactate over 20 minutes. The study drug was calculated according to the weight of the patient, diluted to a total volume of 20 ml, and infused over 15 minutes using a syringe pump at a rate of 80 ml/hour. The anesthesiologist involved in the monitoring of the parameters was blinded to the dose of Dex administered. Hemodynamic parameters (HR, SBP, DBP, and mean arterial pressure (MAP)), SpO2, RR, and Ramsay sedation scores were recorded every five minutes till the infusion was over and subsequently every 15 minutes till the end of surgery.

At the end of the infusion, the patient was placed in the lateral decubitus position, and a subarachnoid block was performed at the L3-L4 interspace using a standard midline approach with a 27- or 26-gauge Quincke needle. Three ml of 0.5% hyperbaric bupivacaine (15 mg) was injected intrathecally for all patients. The patients were then placed in the supine position and given oxygen at 4 L/min via a simple facemask throughout the procedure. Hemodynamic parameters (HR, SBP, DBP, SpO2, and RR) were recorded immediately after intrathecal injection and every two minutes for the first 15 minutes, followed by every five minutes for the rest of the procedure. Sensory blockade was assessed using a cold swab, and motor blockade was assessed using the modified Bromage scale. The highest sensory blockade level and recovery time of both sensory and motor blockades were recorded. Recovery time for sensory blockade is defined as two dermatome regressions from the maximum level achieved, whereas that for motor blockade is the time taken to return to modified Bromage scale 1. The modified Bromage scale is scored as follows: grade 0: no paralysis; grade 1: unable to move hip; grade 2: unable to move hip and knee; grade 3: unable to move hip, knee, and ankle. The duration of analgesia was taken from the time of sensory block achievement until the time of the first request for analgesia. Bradycardia was defined as HR ≤ 50/min and was treated with intravenous glycopyrrolate 0.2 mg. Hypotension was defined as a fall in SBP > 30% of preoperative values <90 mmHg and was treated with 200 ml boluses of crystalloids, and if it persisted, then injection of ephedrine was used.

Sample size calculation

The sample size was calculated based on a two-segment regression of the sensory block of the study by Lee et al. [9]. Assuming a standard deviation of 30.7, a precision of 8, and a confidence interval of 95%, the sample size calculated was 58.

n = (1.96^2^ × 30.7^2^)/8^2^

n = 56.57

We made it 20 per group for easy calculation. We planned to randomize a total of 66 patients because of the expected percentage of approximately 10% of conversion to general anesthesia due to block failure or extended duration of surgery.

Statistical analysis

The data were entered into Microsoft Excel 2007 (Microsoft Corp., Redmond, WA) and analyzed using SPSS version 20 (IBM Corp., Armonk, NY). The data were normally distributed, according to the Kolmogorov-Smirnov test. For descriptive analysis, the categorical variables will be analyzed using frequency and percentages, and the continuous variables will be analyzed by calculating the mean ± standard deviation. For inferential analysis, the numerical data were analyzed using ANOVA, while the categorical data were analyzed using the chi-square test, and a p-value < 0.05 was considered statistically significant.

## Results

This prospective, randomized, double-blind study was conducted on 60 patients, randomized into three groups. The data were entered into Microsoft Excel 2007 and analyzed using SPSS. For descriptive analysis, the categorical variables were analyzed using frequency and percentages, and the continuous variables were analyzed by calculating the mean ± standard deviation. For inferential analysis, the numerical data were analyzed using ANOVA, while the categorical data were analyzed using the chi-square test, and a p-value < 0.05 was considered statistically significant. Group I, group II, and group III patients received intravenous Dex 0.5, 0.75, and 1 mcg/kg, respectively, over 15 minutes just before administration of the subarachnoid block.

The mean age distribution among them was as follows: 41.7 ± 12.6 years, 36.1 ± 10.2 years, and 36.8 ± 12.2 years in groups I, II, and III, respectively (p = 0.2564). There were 13 (65.0%), 12 (60.0%), and 14 (70%) male patients and seven (75%), eight (40%), and six (30%) female patients in groups I, II, and III, respectively (p = 0.803). The weight distribution (in kg) was 64.9 ± 10.5, 71.1 ± 11.9, and 65.7 ± 11.3 in groups I, II, and III, respectively (p = 0.1851). The height distribution (in cm) was 164.6 ± 7.4, 165.2 ± 5.9, and 163.1 ± 5.5 in groups I, II, and III, respectively (p = 0.5517). The BMI distribution was 23.9 ± 3.8, 25.8 ± 3.3, and 24.8 ± 3.4 in groups I, II, and III, respectively (p = 0.2632). Hence, the demographic parameters were comparable between the groups. Also, the duration of surgery (in minutes) was comparable between the groups, i.e., 74.75 ± 24.7, 70.7 ± 26.17, and 71.5 ± 28.5, with a p-value of 0.8796 (Table 1).

**Table 1:** Demographic parameters and duration of surgeries Statistically significant p-value < 0.05.

Demographic parameters and duration of surgeries		Group I, mean ± SD	Group II, mean ± SD	Group III, mean ± SD	F value	P-value
Sex	Male, N (%)	13 (65.0%)	12 (60.0%)	14 (70%)	0.4396	0.803
	Female, N (%)	7 (75.0%)	8 (40.0%)	6 (30.0%)		
Age (years)		41.7 ± 12.6	36.1 ± 10.2	36.8 ± 12.2	1.39	0.2564
Height (cm)		164.6 ± 7.4	165.2 ± 5.9	163.1 ± 5.5	0.60	0.5517
Weight (kg)		64.9 ± 10.5	71.1 ± 11.9	65.7 ± 11.3	1.74	0.1851
BMI		23.9 ± 3.8	25.8 ± 3.3	24.8 ± 3.4	1.37	0.2632
Duration of surgery (minutes)		74.75 ± 24.7	70.7 ± 26.17	71.5 ± 28.5	0.13	0.8796

The two-dermatome regression time (in minutes) and the duration of the motor block (in minutes) were significantly higher in groups II and III when compared to group I, with p-values of 0.0021 and 0.003, respectively. However, the duration of analgesia (time taken to first rescue analgesia) was comparable among the groups (p = 0.7677). Similarly, the Ramsay sedation scores were insignificant between the groups (p = 0.1807) (Table 2). The MAP recorded from five to 140 minutes was statistically insignificant (p-value ranging from 0.938 to 0.126). Also, the heart rate recorded from five to 140 minutes was statistically insignificant (p-value ranging from 0.997 to 0.104).

**Table 2:** Study parameters * Statistically significant p-value < 0.05.

Parameters	Group I, mean ± SD	Group II, mean ± SD	Group III, mean ± SD	F value	P-value
Two-dermatome regression time (in minutes)	107.7 ± 17.2	128.0 ± 15.7	124.2 ± 22.4	6.87	0.0021*
Duration of analgesia (in minutes)	230 ± 52.1	227.2 ± 44.6	239 ± 61.8	0.27	0.7677
Duration of motor block (in minutes)	174.25 ± 20.6	192.5 ± 21.0	206.5 ± 28.0	9.50	0.003*
Ramsay sedation scores	3.33 ± 0.6	3.72 ± 0.6	3.64 ± 0.7	1.76	0.1807

## Discussion

In this study, it was observed that two dermatome regression times and the duration of the motor block during spinal anesthesia were significantly higher with intravenous bolus administration of Dex of 0.75 mcg/kg and 1 mcg/kg, respectively, when compared to 0.5 mcg/kg. The duration of analgesia, sedation scores, and adverse effects were not significantly different between the groups.

Dexmedetomidine, a highly selective α2-adrenergic agonist primarily used as an intravenous sedation, has been found to prolong the block characteristics of both central neuraxial and peripheral nerve blocks, probably due to the central mechanisms [9]. It is also devoid of respiratory depression, unlike other sedatives or opioids. Although many studies are available in the literature that evaluated the effects of intravenous Dex in the subarachnoid block, the dose and method (bolus vs. continuous or both) and the timing (before or after the block) of administration are different. For instance, a few studies administered a bolus dose followed by an infusion [2-7]. Dinesh et al. [2] used a bolus of 1 mcg/kg immediately after the block, followed by an infusion of 0.5 mcg/kg, and compared it with a control group that received saline. They observed that intravenous Dex significantly prolonged the duration of sensory and motor blocks as well as produced a significant incidence of bradycardia requiring atropine. A recent similar study by Bharthi Sekar et al. [3] in patients receiving spinal anesthesia for lower limb and abdominal surgeries also concluded that intravenous dexmedetomidine prolonged the duration of sensory and motor block and also appeared to provide sedation with easy arousability and analgesia postoperatively while maintaining hemodynamic stability with no significant side effects. In contrast, Bhirud et al. [4] used a bolus of 0.5 μg/kg before the block and compared the two different doses of infusion (0.25 vs. 0.5 μg/kg/hr) and observed that the maintenance dose of 0.5 μg/kg/hr was better with stable hemodynamics. In our study, there was also no significant incidence of bradycardia. Ok et al. [5] administered a loading dose of 1 µg/kg after the block, followed by a comparison of the maintenance dose of 0.2 µg/kg/hr vs. 0.4 µg/kg/hr, and concluded that the loading dose was sufficient for surgeries lasting less than 60 minutes, while the infusion of 0.2 µg/kg/hr was sufficient for surgeries lasting 90 minutes. Intravenous Dex 1 mcg/kg as a bolus dose followed by a 0.6 mcg/kg/hr maintenance infusion significantly prolonged sensory and motor blocks, with about one-third of patients requiring atropine for treatment of bradycardia when compared to the placebo group, as per another study [6]. Song et al. [7] concluded that a continuous administration dose of 0.25 µg/kg/hr may be the most appropriate when compared to 0.5 or 0.75 µg after an initial bolus of 1 µg/kg. In contrast to these studies, our study focused only on the efficacy of bolus dose administration to find an optimum dose.

As mentioned in the “Introduction” section, only a few studies compared the effects of different bolus doses of Dex on the subarachnoid block [8-10]. Park et al. [8] concluded that a bolus dose of 1 mcg/kg was superior to 0.5 mcg/kg in the elderly population. In contrast, Lee et al. [9] found that both 1 mcg/kg and 0.5 mcg/kg prolonged the block characteristics with similar incidences of hypotension and bradycardia. Jung et al. [10] found that a bolus dose of 0.5 mcg/kg significantly prolonged the duration of the sensory and motor block when compared to 0.25 mcg/kg with similar hemodynamic effects. However, we observed that both 0.75 mcg/kg and 1 mcg/kg doses produced better block characteristics when compared to the dose of 0.5 mcg/kg.

Using only an infusion of 0.25 μg/kg/hr, started 10 minutes before the block, Zhang et al. [12] observed that this infusion prolonged the duration of sensory and motor blockade when compared to the placebo. Vatsalya et al. [13] found that an infusion of 0.5 μg/kg/hr, following a bolus administration of 0.5 μg/kg, resulted in a significantly prolonged sensory and motor block when compared to the bolus dose alone. In our study, we did not use any continuous infusion after spinal anesthesia, and we believe that it is better to restrict such infusions for cases of long duration. Also, we wanted to focus exclusively on the effects of bolus dose and to confirm precisely that the effects were indeed due to bolus dose only. In a review article by Al Nobani et al. [16], they concluded that considering both advantages and disadvantages, the use of a 1 mcg/kg loading dose of dexmedetomidine was associated with a larger side-effect profile, while the beneficial changes to the characteristics of the subarachnoid blockade were minimal when compared to lower loading doses, and in that sense, a lower loading dose should be preferred.

The main limitation of our study is that a control group receiving a placebo could not be added as the IRB objected to it. We observed that the two dermatome regression times and the duration of the motor block were significantly higher with 0.75 mcg/kg and 1 mcg/kg when compared to 0.5 mcg/kg. We suggest that it is better to titrate the dose between 0.75 and 1 mcg/kg to suit the individual needs.

## Conclusions

Bolus administration of intravenous Dex in doses of 0.75 and 1 mcg/kg when compared to 0.5 mcg/kg could prolong the two-dermatome regression time of sensory block and the duration of motor block significantly. However, there was no difference in terms of duration of analgesia, time taken to first rescue analgesia, Ramsay sedation scores, or adverse events between the groups. Therefore, administration of bolus intravenous Dex in doses ranging between 0.75 and 1 mcg/kg appears to provide adequate intraoperative block characteristics while maintaining hemodynamic stability without any significant respiratory depression or other adverse effects.
